# Mental health and inclusivity support and education in a UK dental school: a cross-sectional survey

**DOI:** 10.1038/s41415-022-5311-6

**Published:** 2022-12-16

**Authors:** Chloe Lennox, Jay-Krishan Pandya, Ross Lyttle, Sohum Pandya, Chris Penlington, Charlotte Bowes

**Affiliations:** School of Dental Sciences, Newcastle University, Newcastle, NE2 4AZ, UK

## Abstract

**Aims ** To establish the current support and knowledge around mental health and equality, diversity and inclusion at a UK Dental School and make suggestions about improvements.

**Objectives ** Conduct a survey of dental undergraduate students at Newcastle Dental School to elicit responses

**Methods** Bachelor of Dental Surgery and Bachelor of Oral and Dental Health Science students at Newcastle University were invited to participate in this cross-sectional survey via email. Following electronic consent, an electronic questionnaire via an online form builder was distributed via email. Participants were asked questions on experiences and feelings towards wellbeing support, mental health and equality, diversity and inclusion topics, including improvements that could be made.

**Results ** In total, 89 students participated. The majority of participants were white, women and heterosexual. Wellbeing support was present and generally accessible. Students were aware of mental health conditions but unsure how to manage them in a clinical setting. Students were generally unaware of barriers to care faced by LGBT+ and racialised minority patients. Students were mainly uncomfortable disclosing personal issues with their personal tutor. Students responded positively to some suggested improvements in support and education surrounding mental health and inclusivity.

**Conclusion ** Our study highlighted the areas where Newcastle Dental School continues to provide high levels of support for students but also areas that may require attention through further study and focus groups, with an aim to increase diversity of respondents so that further exploration regarding the intersectionality of identity can be undertaken.

**Supplementary Information:**

Zusatzmaterial online: Zu diesem Beitrag sind unter 10.1038/s41415-022-5311-6 für autorisierte Leser zusätzliche Dateien abrufbar.

## Key points

- Raises awareness of mental health problems and equality, diversity and inclusion issues faced by both professionals and patients.
- Identifies areas of improvement to mental health and equality, diversity and inclusion education and support within Newcastle University School of Dental Sciences which could be looked at within other dental schools.
- Led to actions taken within the School of Dental Sciences to address issues which could be used as an example in other professional settings.

**Supplementary Information:**

Zusatzmaterial online: Zu diesem Beitrag sind unter 10.1038/s41415-022-5311-6 für autorisierte Leser zusätzliche Dateien abrufbar.

## Introduction

Throughout this study, the term mental health (MH) has been used as an umbrella term referring to mental wellbeing and mental health problems. The terms racialised minorities (RM) and LGBT+ have been used throughout the project; however, we recognise that neither of these communities are homogenous groups. As part of this project, 'support' encompasses management of MH and equality, diversity and inclusion (EDI)-related problems, including counselling and signposting to appropriate services.

One in six people over the age of 16 report having a MH problem,^1^ with men accounting for three-quarters of suicide-related deaths in 2020 in England and Wales.^2^ Dentistry is a stressful career, with burnout within the profession being common; a study in the *European Journal of Dental Education* found that before the COVID-19 pandemic, around 40% of clinical dental and dental hygiene and therapy students in one university met the criteria for burnout.^3^ Occupational stress is one of the leading causes of MH problems within the dental profession, with 'job strain' and 'effort reward imbalance' being identified as major culprits.^4^

The COVID-19 pandemic has led to numerous physical, mental, financial and academic concerns for students, with a survey by Mind finding that just over one-third (34%) of young people in England and Wales stated that their MH had worsened since the pandemic began.^5^ Additionally, a survey of UK dental students found that 74% of respondents felt that the COVID-19 pandemic had increased their need for wellbeing support, with suspension of university activities and transitioning to online learning contributing to this rise.^6^ A survey of dentists in Wales post COVID-19 pandemic found that 82% of respondents reported a noticeable increase in stress levels among the dental team.^7^

Dental professionals treat patients who may have MH diagnoses and confide in us. As one in four adults think that they have a MH condition at some point in their life,^8^ education about different MH issues, where to access support (such as counselling) and how to prioritise both patient and personal wellbeing can only be welcomed. The impact of MH conditions on oral health have been widely researched, with antidepressant and antipsychotic medication potentially leading to xerostomia, dietary changes and reduction in oral hygiene practices. Multiple oral complications have been associated with eating disorders, such as erosive tooth surface loss, traumatic lesions within the mouth and oral health consequences of malnutrition.^9^

Evidence suggests that RM may be more likely to experience MH issues compared to their White British counterparts, with African Caribbean individuals being three to five times more likely to be diagnosed and admitted to hospital for schizophrenia. This disparity may be a result of lack of understanding and stigma towards and within these cultures and ethnicities.^10^

RM student populations have reported a lack of RM-specific curricula content, resulting in discomfort and isolation.^11^ Provision of a more inclusive curriculum and environment may aid in reducing this gap. RM comprise 13.8% of the UK population^12^ and 35% of General Dental Council (GDC) registrants are from minority ethnic groups.^13^ It is vitally important to ensure the needs of the RM community are catered to. For example, fit-testing for FFP3 masks during the COVID-19 pandemic was required, with guidance stating men should be clean-shaven; however, this may not always be possible among some religious communities.^14^ Further, individuals of RM face multiple barriers to accessing healthcare services, including a lack of inclusivity due to language and communication barriers and possible feelings of discrimination.^15^ As a result of these barriers, they may be less likely to attend dental services for routine appointments and are more likely to only attend for urgent dental care.^15^ Dietary requirements also often differ between ethnic groups, which dental professionals should be cognisant of when providing oral health and dietary advice.^16^

The LGBT+ community is growing and as such, it is important for dental professionals to improve their awareness of specific health needs and reduce discrimination this community may face. In 2018, a YouGov survey with 5,000 LGBT+ individuals across England, Scotland and Wales found that just over 50% of this population reported experiencing depression in the last year, stating that rejection from family and friends, harassment and experience of hate crimes negatively impacted their mental wellbeing.^17^ Research shows that LGBT+ individuals continue to face barriers to health care today because of discrimination and lack of understanding from healthcare professionals of their specific health needs when accessing services. One in eight LGBT+ people have experienced unequal treatment from healthcare staff, which is likely to result in avoidance of health services.^17^

There is a current drive for the GDC to champion EDI in the coming years which extends to both patients and staff.^18^

## Aims

- To explore the perceived current level of support regarding personal MH and EDI issues within Newcastle University (NU) (including access to counsellors, personal tutor system and feelings of inclusivity from staff, colleagues and patients) for Bachelor of Dental Surgery (BDS) and Bachelor of Science in Oral and Dental Health Sciences (ODHS) students
- To explore BDS and ODHS student knowledge of MH and EDI issues pertaining to student-patient clinical interactions
- To suggest areas for improvement pertaining to MH and EDI in NU School of Dental Sciences (SDS).

## Methodology

A cross sectional survey of undergraduate BDS and ODHS students from NU was undertaken. Ethical approval was obtained through NU (Ref: 2027/6903).

### Participant recruitment

An electronic survey, together with participant information and consent form, was internally distributed via email to all BDS and ODHS students. The survey remained open for 24 days. A reminder for survey completion was automatically sent to those who had not completed the survey one week before survey closure. There was opportunity to ask questions of the research team before participation.

### Survey design

The survey was designed by dental students and was checked by staff members to ensure questions were understandable and appropriate to the research question. The survey was tested among several dental student colleagues before going 'live'. The questionnaire probed student knowledge of depression, anxiety and dental phobia due to their high prevalence in the UK and widely known oral health impacts.^9^^,^^19^ Other MH disorders incorporated in the survey (such as bipolar disorder and schizophrenia) were included due to their higher prevalence (in the UK) compared to other disorders.^19^

### Data collection

Data were collected from December 2020 to January 2021. After completing electronic written consent, participants were immediately taken to the questionnaire. An online survey building platform was used (Jisc 2020). The questionnaire collected participant demographic information; student ratings of knowledge and confidence to manage patients presenting with MH issues and patients from RM and LGBT+ communities; and student experiences and feelings towards availability of wellbeing support in SDS and any improvements that could be made to improve access and efficacy of services. Tables 1, 2, 3 and 4 show survey questions which were a combination of dichotomous yes/no answers and a rating scale (0-4).

**Table 1:** Number and percentage of respondents for dichotomised survey questions

**Survey questions**	**n (%)**	
	**Yes**	**No**
Are you aware of wellbeing services at NU? (n = 89)	59 (67.0)	29 (33.0)
Have you accessed NU wellbeing services? (n = 89)	24 (27.6)	63 (72.4)
If yes, were you able to access with ease? (n = 24)	20 (83.3)	4 (16.7)
Are you aware of wellbeing service information on Canvas? (n = 89)	25 (28.7)	62 (71.3)
Are you aware of British Dental Association's Health Assured, which is a counselling and emotional support service for dental professionals and students? (n = 9)	18 (20.9)	68 (79.1)
If no, interested in more information? (n = 68)	56 (82.4)	12 (17.6)
Ever accessed support from an external wellbeing service? (n = 89)	24 (27.3)	64 (72.7)
Have you had any experience treating patients with mental health concerns? (n = 51)	38 (74.5)	13 (25.5)
Are you aware of where to signpost patients with mental health concerns if this is required? (n = 51)	8 (15.7)	43 (84.3)
Do you know when it is appropriate to step in regarding a patient's mental health? (n = 51)	12 (24.5)	37 (75.5)
With regards to personal protective equipment, religious/cultural beliefs catered to? (n = 89)	65 (90.3)	7 (9.7)
Have you ever felt uncomfortable speaking up regarding LGBT+? (n = 89)	12 (15.0)	68 (85.0)

**Table 2:** Number and percentage of respondents for scaled survey questions

**Survey question (ranked 0-4: 0 = worst, that is, not informed/least satisfied; 4 = best, that is, very informed/very satisfied)**	**n (%)**				
	**0**	**1**	**2**	**3**	**4**
How well informed do you feel regarding where to seek support concerning your mental health at NU?	4 (4.5)	12 (13.5)	29 (32.6)	27 (30.3)	17 (19.1)
In general, how easy is it for you to be open about issues or difficulties you may be having?	12 (13.6)	29 (33.0)	2 (22.7)	21 (23.9)	6 (6.8)
How comfortable would you feel about discussing difficulties with your personal tutor?	9 (10.1)	24 (27.0)	23 (25.8)	19 (21.3)	14 (15.7)
How satisfied have you been with your experience of the wellbeing support services? (n = 24)	2 (8.3)	3 (12.5)	5 (20.8)	4 (16.7)	10 (41.7)
How comfortable would you be discussing and raising a concern for a patient's mental health with a clinical supervisor? (n = 51)	2 (3.9)	3 (5.9)	10 (19.6)	20 (39.2)	16 (31.4)
Generally, how confident are you that your cultural/religious beliefs have been catered to in Newcastle Dental School? (n = 69)	2 (2.9)	6 (8.7)	9 (13.0)	12 (17.4)	40 (57.9)
How confident are you providing advice to patients with different cultural/religious beliefs?	16 (20.0)	24 (30.0)	16 (20.0)	11 (13.8)	13 (16.3)
How welcoming do you think the school is to students, staff, patients and visitors who are LGBT+?	0 (0)	3 (3.7)	10 (12.3)	19 (23.5)	49 (60.5)
How informed do you feel regarding LGBT+ barriers to health care?	17 (20.7)	17 (20.7)	27 (32.9)	14 (17.1)	7 (8.5)

**Table 3:** Number and percentage of respondents for scaled survey questions

**Question**	**n (%)**		
**How well informed are you about each of these MH conditions?**	**Very informed**	**Somewhat informed**	**Not informed**
Dental phobia	30 (58.8)	21 (41.2)	0 (0)
Generalised anxiety	17 (33.3)	34 (66.7)	0 (0)
Depression	12 (32.5)	36 (70.6)	3 (5.9)
Eating disorders	6 (11.8)	30 (58.8)	15 (29.4)
Schizophrenia	0 (0)	15 (29.4)	36 (70.6)
Bipolar disorder	1 (2.0)	15 (29.4)	35 (68.6)
**How often do you ask patients about mental health problems?**	**Always**	**Sometimes**	**Never**
	44 (86.3)	6 (11.8)	1 (2.0)

**Table 4:** Number and percentage of respondents for scaled survey questions

**Suggestions for improvement**	**n (%)**		
	**Beneficial**	**Unsure**	**Not beneficial**
Professional mental health advisor for dental/medical students	84 (94.2)	4 (4.5)	1 (1.1)
Student wellbeing representative	66 (74.2)	15 (16.9)	8 (9.0)
Training on mental health first aid/suicide prevention	79 (89.8)	8 (9.1)	1 (1.1)
Wellbeing/resilience training incorporated into the curriculum	72 (80.9)	13 (14.6)	4 (4.5)
Implement mental health awareness day or at least some dedicated time incorporating equality and diversity, racism and LGBT+	63 (70.8)	16 (18.0)	10 (11.2)
Increased education around different cultures and beliefs	73 (82)	11 (12.4)	5 (5.6)
Communication skills session on how to handle racist comments	81 (91.0)	5 (5.6)	3 (3.4)

### Data analysis

Data were anonymised, cleaned and the accuracy checked by the research team. Descriptive statistics were used to explore the data. The proportion of participants that answered each question is broken down in Tables 1, 2, 3 and 4.

## Results

In total, 89 participants consented and completed the questionnaire. A response rate of 20.5% (89 out of 435 students) was achieved. Respondents were, in the majority, BDS students (93.3%), women (76.4%) and heterosexual (80.9%). The majority of respondents were of white ethnicity (71.9%) and 51.9% participants reported having religious beliefs. Participant demographics can be seen in Table 5.

**Table 5:** Participant demographics collected

**Variable**	**Characteristic**	**Number (n)**	**Percentage (%)**
Course studying	BDS	83	93.26
	ODHS	6	6.74
Sex	Female	68	76.40
	Male	21	23.60
Sexual orientation	Heterosexual	72	80.90
	Homosexual	5	5.62
	Bisexual	8	8.99
	Prefer not to say	4	4.49
Ethnicity	**White ethnic groups**	64	71.91
	White English/Scottish/Welsh/Northern	62	69.66
	White Irish	1	1.12
	White other background	1	1.12
	**Black/African/Caribbean/Black British groups**	2	2.25
	Black African	2	2.24
	**Asian/Asian British ethnic groups**	16	17.98
	Indian	5	5.62
	Pakistani	5	5.62
	Bangladeshi	1	1.12
	Chinese	2	2.25
	Other Asian background	3	3.37
	**Multiracial ethnic groups**	6	6.74
	White and Black Caribbean	1	1.12
	White and Asian	3	3.37
	Any other multiracial ethnic background	1	1.12
	**Other ethnic groups**	2	2.25
	Arab	1	1.12
	Other	1	1.12
Religious beliefs	No religion	43	48.31
	Christian	27	30.34
	Buddhist	1	1.12
	Hindu	3	3.37
	Muslim	9	10.11
	Sikh	1	1.12
	Other (agnostic)	1	1.12
	Prefer not to say	4	4.49
Clinical or pre-clinical	Clinical	51	57.30
	Pre-clinical	38	42.70

In total, 27% percent of participants reported they had accessed support from NU's wellbeing services, of which, 58.4% of students reported being satisfied with this service. Of the survey participants, 27% reported that they had accessed support from an external wellbeing service; it is unclear whether these participants had also accessed internal services. Over one-third (37.1%) of participants reported that they would not feel comfortable discussing difficulties with their personal tutor.

The majority of participants were clinical students (57%), of which, 74.5% had experience of treating patients with MH problems, with 86.3% asking about MH at each medical history-taking episode. Almost three-quarters (72.5%) of clinical participants were unaware of when it is appropriate to raise a concern regarding a patient disclosure of a MH problem and 84.3% were unaware of where to signpost patients. Over 70% felt comfortable discussing and raising a patient MH concern with a clinical supervisor.

More than half of participants (57.4%) felt confident that their cultural and religious beliefs are catered to; 11.6% reported not being confident, of which, 25% were Muslim. Of all participants, 50% stated that they were not confident in providing dental care and advice to patients with different cultural/religious beliefs.

A proportion of participants (13.5%) reported that they have felt uncomfortable speaking up on LGBT+ issues because of fear of negatively impacting upon patient rapport. One-third of the respondents who felt uncomfortable speaking up described their sexual orientation as homosexual or bisexual. Participants felt less informed on LGBT+ barriers to health care, with 41.2% saying they were uninformed.

## Discussion

The data we chose to report on reflects the areas where we felt improvements could be proposed within NU SDS. Data that we have not discussed in detail is reflective of student overall satisfaction with these areas and felt they did not require much elaboration as per the aim of the study.

### Mental health

The majority of students reported being aware of how and where to access wellbeing support and services at NU, suggesting that there may be adequate signposting and knowledge of how to access support services. A minority of participants reported they were dissatisfied with the wellbeing services. Although we did not collect data as to why this was, anecdotal evidence suggests that this may be due to perceived long waiting lists, unsuitable appointment timings or a limit to the number of sessions available. These disadvantages of university wellbeing services could have led to students seeking external support. A close correlation between longer wait times in MH services, with its impact on patients and therefore poorer health outcomes, has been noted,^20^with two-fifths of patients on the waitlist for MH services forced to resort to emergency services.^21^

The personal tutor scheme at NU, whereby students are paired with an academic or clinical staff member, aims to provide support for the student body; however, most students reported feeling uncomfortable around sharing a personal problem with them. As the personal tutor at NU should be the first point of contact for students who are struggling, it is important to understand why this might be the case and further research is indicated.

Participants in clinical years of the course reported feeling uninformed about eating disorders and depression, which are highly relevant to oral health. Dental phobia and anxiety were conditions that students felt most informed about; however, many students felt less informed on conditions such as schizophrenia and bipolar disorder. A close relationship between psychological status, dental phobia and dental avoidance has been found.^22^ Dental avoidance results in poorer oral health, predisposing patients to an increased risk of caries, periodontal disease and oral cancer.^23^ It is therefore important that dental professionals are appropriately educated in MH conditions, both at undergraduate level and in foundation training, to enable professionals to recognise the impact of MH conditions on oral health and have the confidence to signpost and raise a concern if required.

### Racialised minorities

Some participants felt their cultural/religious beliefs were not catered to within the SDS. This question did not allow for free text. It would be interesting to further explore reasons behind feelings of these students. Less than one-third of students felt confident in providing appropriate and tailored dental care and advice to patients with differing cultural/religious beliefs and practices. The importance of this has been highlighted through research assessing the dietary habits and cultural practices of mothers who are Orthodox Jews in North London; many cultural and religious ceremonies involve increased consumption of sugary foods, which increases their risk of dental caries.^24^ Further, the incidence of oral cancer has been found to be higher in people from South Asian backgrounds, linked with the common practice of chewing betel nut.^25^ Teaching into the common diverse practices and beliefs would empower dental professionals to provide tailored oral hygiene and dietary advice while aligning with patient culture and religion. Within medical education, cultural competence (CC) is a model used to address race and culture as social determinants of health, enabling students to better understand race and cultures. Research suggests that integration of CC curricula using a combination of online training, community engagement and reflection may increase the cultural knowledge and skills of dental students. This helped to inform our suggestion of further EDI training and events for staff and students and appointing EDI representatives to deliver projects enhancing the education on these topics.^26^

### LGBT+

One in seven LGBT+ patients avoid seeking health care from fear of being discriminated against and one in eight have experienced unequal treatment from healthcare staff.^14^ Despite this, most of the participants reported feeling uninformed on LGBT+ barriers to health. However, as the majority of respondents identified as heterosexual, they are unlikely to have had the same experiences of LGBT+ issues as those of people of marginalised sexual orientations and gender identities. Therefore, education around LGBT+ barriers to health care are necessary, with the aim to improve access to health care, along with increasing inclusivity for dental team members. Education has shown to be effective in increasing the comfort of LGBT+ patients but also increasing confidence of students providing their treatment.^27^ Consideration of the recommendations by Stonewall to medical schools for reducing barriers to health services for LGBT+ individuals helped us to formulate an action plan of improving education on reducing barriers to health care as a dental profession. Stonewall's recommendations included a review into the curricula, standards and training to cover homophobic language and providing more inclusive care.^17^

### Study limitations

Of the 419 students enrolled in the BDS and ODHS programmes collectively, only 89 students participated in the study. The low response rate has the potential to bias our results due to inherent differences between respondents and non-respondents. This is due to the majority of respondents being white, women and heterosexual, which has limited representation of the intersectionality of identity. Homogeneity of the survey sample is unlikely to reflect the population of BDS and ODHS students and therefore conclusions drawn from this study are not necessarily generalisable.

Despite the number of those suffering with MH problems today, there is still stigma attached to them. However, research shows that RM have more stigma for common MH disorders when compared with majorities, with RM perceiving someone with MH illness as more dangerous than their white counterpart.^28^ This stigma may begin to explain the low response rate from those of RM in this research.

### Outcome and further work

Newcastle SDS has been engaging and encouraging of our research and eager to involve the student body to improve MH support and education. Changes implemented by SDS are shown in online Supplementary Table 1.

## Conclusion

MH and EDI have great relevance to dental professionals and therefore education, awareness and support should be made readily available to students of the profession. Increasing accessibility of student wellbeing services proves crucial in reducing the stigma around MH. Education around MH conditions, LGBT+ barriers to health care and cultural diversity will aid in reducing inequalities for students, staff and patients by improving knowledge and comfort around varying scenarios dental students may encounter in their professional lives. This study has helped to identify gaps within education and knowledge of MH and EDI topics and has led to key changes within SDS.

## Helplines and support services

If you or anyone you know has been impacted by the sensitive topics discussed within this paper, please ask for help. Below are a number of helplines and support services for members of the dental team.

### For urgent and immediate support

- For a life-threatening crisis: 999
- NHS 111
- Samaritans UK: 116-123.

### For general mental health and wellbeing support

- Your own general practitioner
- Occupational Health
- NHS people: 0300-131 7000 (available at https://people.nhs.uk/)
- BDA Health Assured (available at ).

## Supplementary Information

Supplementary Table 1 (PDF 151KB)
