# Supplementary material for: Mental health and inclusivity support and education in a UK dental school: a cross-sectional survey
Source: Br Dent J. 2022 Dec 16;233(12):1029–34. doi: 10.1038/s41415-022-5311-6 (PMC9756726; doi:10.1038/s41415-022-5311-6)
Supplement: Supplementary file 1 — Supplementary Table 1 (PDF 151KB) [file 41415_2022_5311_MOESM1_ESM.pdf]

	Implemented change
1	Introduction of posters with QR codes taking students directly to the support services website placed around common student areas in SDS (designed by students in conjunction with student wellbeing services)
2	Review of the school's virtual learning environment is being reviewed by students to suggest changes to format/layout to make access to wellbeing resources more intuitive
3	NHS pride badges along with information cards have been made available to all staff and students in Newcastle Dental Hospital
4	Creation of a student Welfare Officer role within the Dental Student's Society Committee
5	Employment of a dedicated student wellbeing advisor to work in the school
6	Appointment of EDI student representatives involved in organising and delivering projects to enhance education on EDI topics
7	Provision of further EDI training for students and staff members including interactive student-led EDI introductory sessions for first year BDS and ODHS students
8	Modification to the raising concerns policies to include clear and explicit guidance for reporting EDI issues and accessing local support
9	Decolonisation and diversification of the dental curriculum
10	Hosting of multicultural events along with invited speakers to enhance learning opportunities for students and staff

Supplementary table 1: changes implemented by SDS following study
